# Supplementary material for: Identification and genetic diversity analysis of a male-sterile gene (MS1) in Japanese cedar (Cryptomeria japonica D. Don)
Source: Sci Rep. 2021 Jan 15;11:1496. doi: 10.1038/s41598-020-80688-1 (PMC7810747; doi:10.1038/s41598-020-80688-1)
Supplement: Supplementary file 1 — Supplementary Information. [file 41598_2020_80688_MOESM1_ESM.pdf]

# **Identification and genetic diversity analysis of a male-sterile gene (*MS1*) in Japanese cedar (*Cryptomeria japonica* D. Don)**

Yoichi Hasegawa[1$], Saneyoshi Ueno[1*], Fu-Jin Wei[1$], Asako Matsumoto[1], Kentaro Uchiyama[1], Tokuko Ujino-Ihara[1], Tetsuji Hakamata[2], Takeshi Fujino[3], Masahiro Kasahara[3], Takahiro Bino[4], Katsushi Yamaguchi[4], Shuji Shigenobu[4], Yoshihiko Tsumura[5], Yoshinari Moriguchi[6*]

1 Department of Forest Molecular Genetics and Biotechnology, Forestry and Forest Products Research Institute, Forest Research and Management Organization, Tsukuba, Ibaraki, Japan.

2 Forestry and Forest Products Research Center, Shizuoka Prefectural Research Institute of Agriculture and Forestry, Hamamatsu, Shizuoka, Japan.

3 Graduate School of Frontier Sciences, The University of Tokyo, Kashiwa, Chiba, Japan

4 National Institute for Basic Biology, Okazaki, Aichi, Japan.

5 Faculty of Life and Environmental Sciences, University of Tsukuba, Tsukuba, Ibaraki, Japan.

6 Graduate School of Science and Technology, Niigata University, Niigata, Niigata, Japan.
