# Supplementary material for: Identification and genetic diversity analysis of a male-sterile gene (MS1) in Japanese cedar (Cryptomeria japonica D. Don)
Source: Sci Rep. 2021 Jan 15;11:1496. doi: 10.1038/s41598-020-80688-1 (PMC7810747; doi:10.1038/s41598-020-80688-1)
Supplement: Supplementary file 2 — Supplementary Figure S1. [file 41598_2020_80688_MOESM2_ESM.pdf]

Haplotypes	Wild Type Ajigasawa20_1	*ms1* Ooi-7_1 (30bp deletion)
Peptide sequence	MAAIVRALIIFVLMAVAESVMTQQTDAAIDCTDPIVSLSPCLSYMTRSAKLSIPDDDC CDSLATVINTHAGCLCELVSRDDFLGFPINQTITLSLPSTCDIQYPRELDQCIVVLPPSS DGPVVQPGSNPPPRLFHPEAPSPALEAVPPVLEAPPMESVPDQPDDSPFPNVSIAG ALFTQSLLRLFWGSLLTAILTVGYLH	MAAIVRALIIFVLMAVAESVMTQQTDAAIDCTDPIVSLSPCLSYMTRSAKLSIPDDDC CDSLATVINTHAGCLCELVSRDDFLGFPINQTITLSLPSTCDIQYPRELDQCIVVLPPSS DGPVVQPGSNPPPRLFHPEAPSPALEAVPPVLEAPPMESVPDQPDDSPFPNVSIAG ALFTQSLLRLFWGSLH
Prediction of potential C-terminal GPi-modification site	Potential GPI-modification site was found.<br>Quality of the site .... : S<br>Sequence position of the omega-site : **176**<br>Score of the best site .... : -3.28 (PValue = 2.791270e-03)	**None** potential GPI-modification site was found.<br>Among all positions checked, sequence position 160 had the best score.

Figure S1 Results of a 30-bp deletion on the loss of potential GPI-modification sites. Peptide sequences of Ajigasawa20_1 and Ooi-7_1 were tested for prediction of potential GPI-modification site using big-PI Plant Predictor (Eisenhaber *et al.* 2003).

Reference

Eisenhaber, B. *et al.* Glycosylphosphatidylinositol lipid anchoring of plant proteins. Sensitive prediction from sequence- and genome-wide studies for Arabidopsis and rice. *Plant Physiol.* **133,** 1691–1701 (2003).
