# Supplementary figures and images for: Identification and genetic diversity analysis of a male-sterile gene (MS1) in Japanese cedar (Cryptomeria japonica D. Don)
Source: Sci Rep. 2021 Jan 15;11:1496. doi: 10.1038/s41598-020-80688-1 (PMC7810747; doi:10.1038/s41598-020-80688-1)

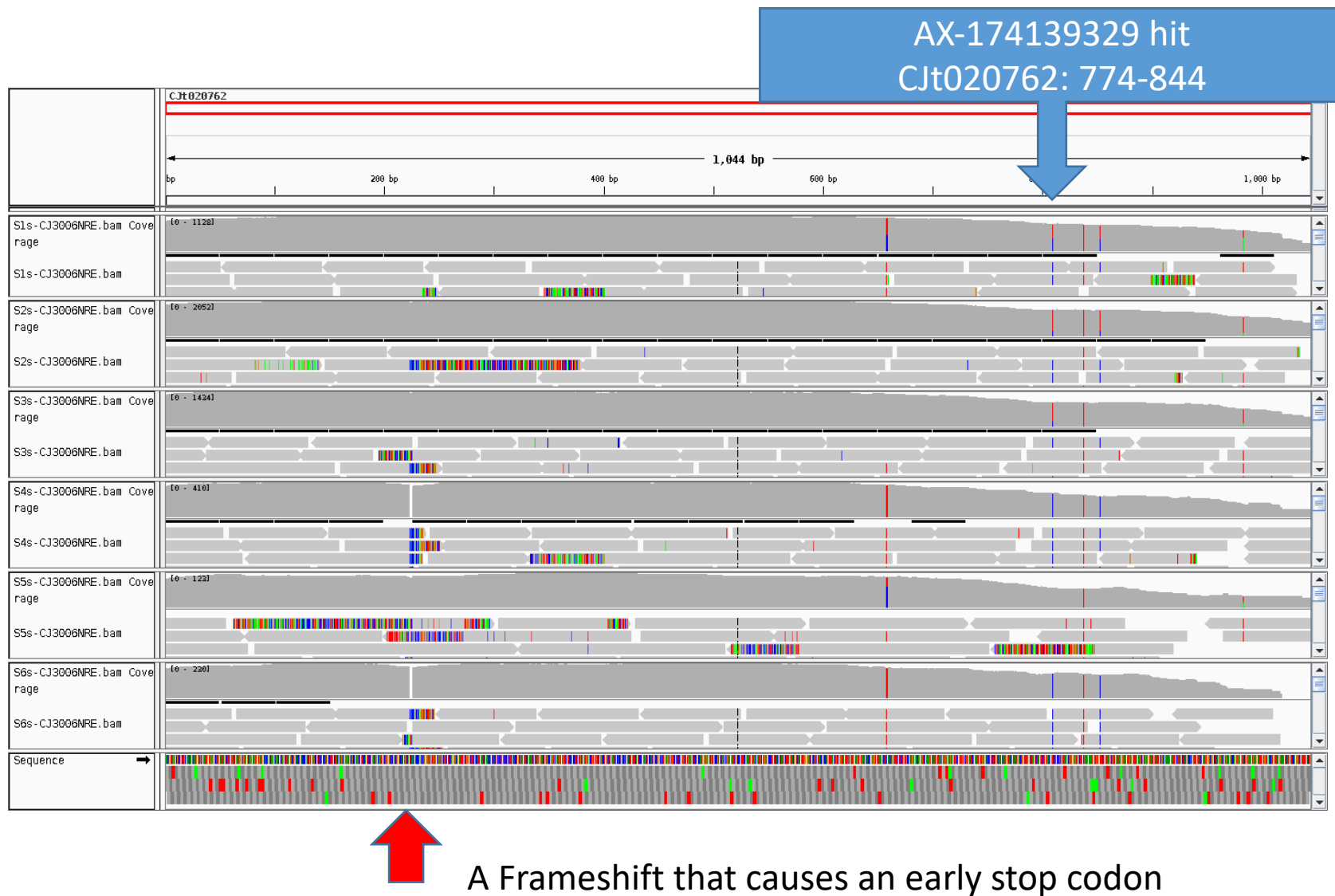

Figure S2 A 4bps deletion and a SNP marker in CJt020762.

Supplement: Supplementary file 3 — Supplementary Figure S2. [file 41598_2020_80688_MOESM3_ESM.pdf]

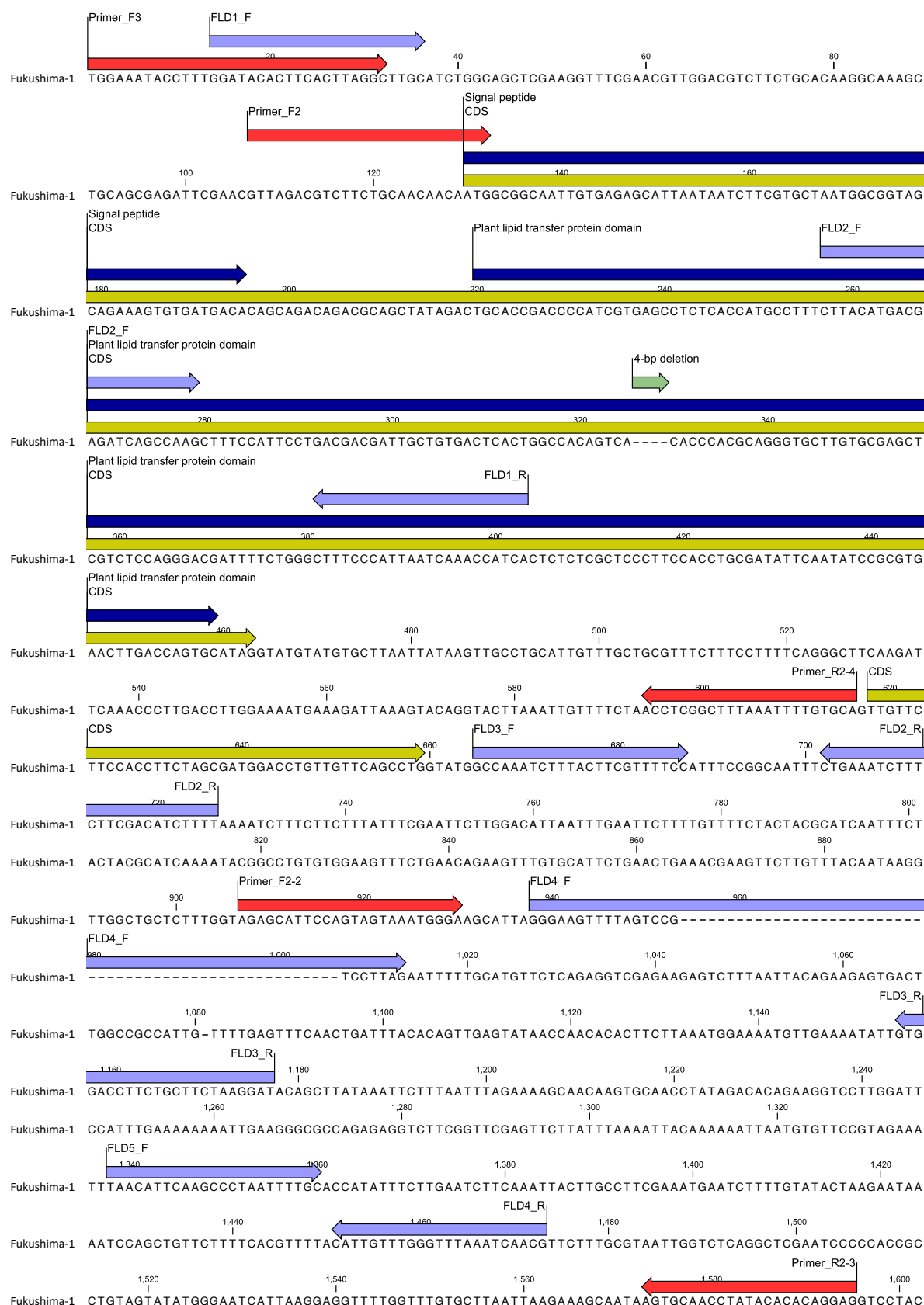

Figure S3 Genomic DNA sequence of CJt020762 for Fukushima-1 and primer site position.

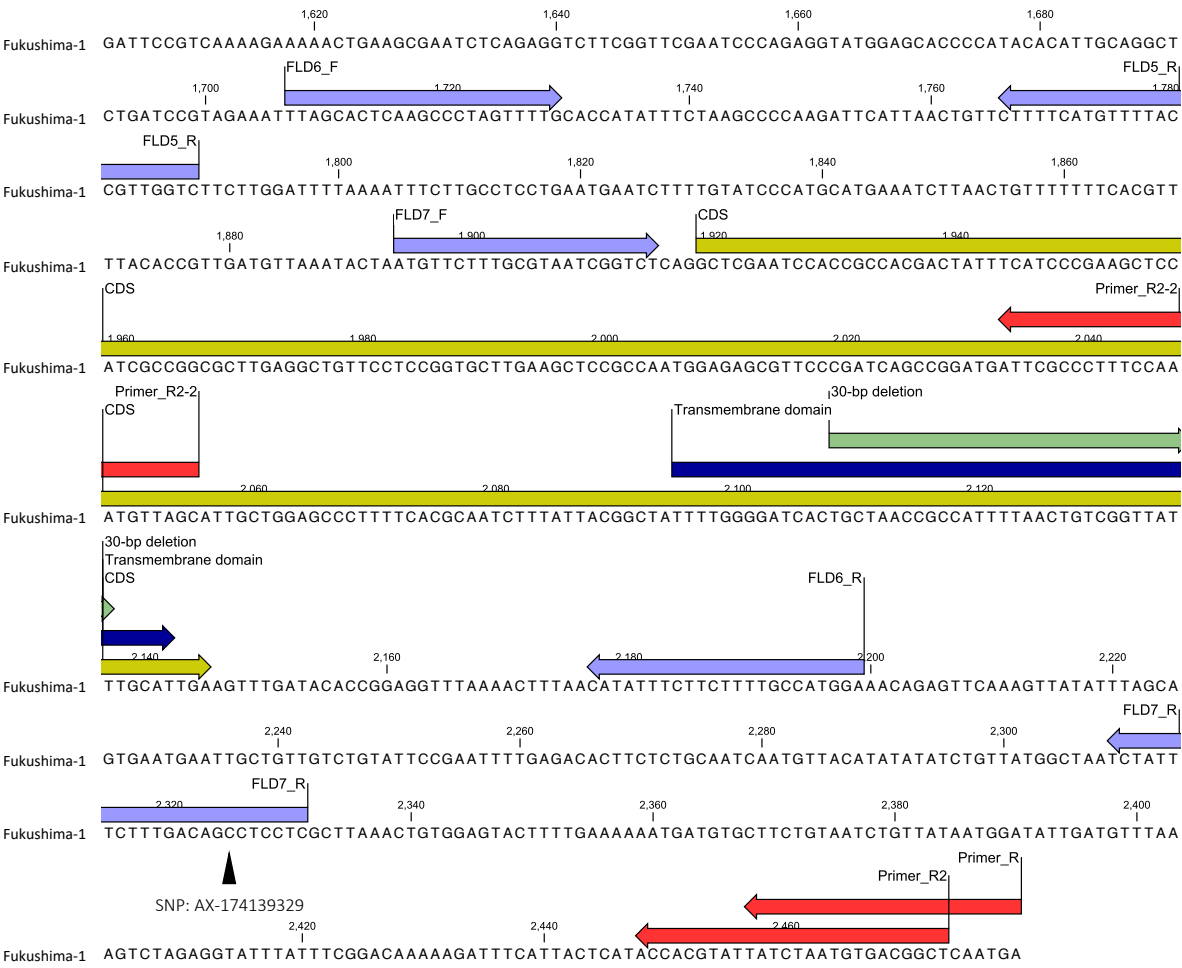

Figure S3 (continued)

Supplement: Supplementary file 4 — Supplementary Figure S3. [file 41598_2020_80688_MOESM4_ESM.pdf]
